# Molecular Mechanisms of Action of Dendrimers with Antibacterial Activities on Model Lipid Membranes

**DOI:** 10.3390/polym17070929

**Published:** 2025-03-29

**Authors:** Albena Jordanova, Asya Tsanova, Emilia Stoimenova, Ivan Minkov, Aneliya Kostadinova, Rusina Hazarosova, Ralitsa Angelova, Krassimira Antonova, Victoria Vitkova, Galya Staneva, Ivo Grabchev

**Affiliations:** 1 Faculty of Medicine, Sofia University “St. Kliment Ohridski”, 1 Koziak Street, 1407 Sofia, Bulgaria; assja_t@yahoo.com (A.T.); ekstoimeno@uni-sofia.bg (E.S.); minkov.ivan@gmail.com (I.M.); i.grabchev@chem.uni-sofia.bg (I.G.); 2 Rostislaw Kaischew Institute of Physical Chemistry, Bulgarian Academy of Sciences, Acad. G. Bonchev Str., Bl. 11, 1113 Sofia, Bulgaria; 3 Institute of Biophysics and Biomedical Engineering, Bulgarian Academy of Sciences, Acad. G. Bonchev Str., Bl. 21, 1113 Sofia, Bulgaria; aneliakk@yahoo.com (A.K.); r_hazarosova@abv.bg (R.H.); angelova@abv.bg (R.A.); 4 Institute of Solid State Physics, Bulgarian Academy of Sciences, 72 Tsarigradsko Shose Blvd., 1784 Sofia, Bulgaria; krasa@issp.bas.bg (K.A.);

**Keywords:** dendrimers, POPC, monolayers, liposomes

## Abstract

In the last decades, numerous dendrimers with a variety of potential biomedical applications have been developed and investigated. The aim of the present study was to evaluate the molecular mechanisms of interaction between two dendrimers with proven antibacterial activity (4-*N*,*N*-dimethylamino-1,8-naphthalimide (Dab) and 3-bromo-Dab (Dab-Br)) and POPC (1-palmitoyl-2-oleoylphosphatidylcholine) model membranes (monolayers and liposomes). The pressure-area isotherms and the compressional modulus of the monolayers revealed that Dab is likely to penetrate the hydrophobic region of POPC, whereas Dab-Br inserts mainly into the lipid headgroup area. This assumption was confirmed by FTIR-ATR of POPC liposomes containing Dab and Dab-Br dendrimers. In addition, Dab induced a higher lipid order in POPC large unilamellar vesicles (LUVs) compared to Dab-Br. Moreover, both dendrimers changed the negative zeta potential of POPC vesicles to positive values, with slightly higher effect of Dab-Br, indicating electrostatic interactions between the lipid headgroups and dendrimers. Furthermore, Dab was able to reduce the average POPC LUVs’ size, unlike Dab-Br. The visualization of giant unilamellar vesicles revealed that the increasing dendrimer concentration induced model membrane shrinking and complete disintegration, which was more prominent for Dab. Based on the experimental results, new fundamental knowledge about the destabilizing effect of dendrimers on model lipid membranes will be acquired with a focus on their application in pharmacology and clinical practice.

## 1. Introduction

Infectious diseases caused by various types of ubiquitous pathogens (viruses, bacteria, protozoa, and fungi) are one of the leading causes of death in the world. Over the past decades, the progress of modern medicine has been associated with the discovery and application of effective antibacterial and antifungal drugs that inhibit the growth of infectious agents [1,2,3,4,5]. The widespread and unreasonable use of antibiotics in clinical practice in recent decades, especially during the last SARS-CoV-2 pandemic, has led to the development of pathogen resistance, which is one of the most serious public health problems globally [6,7]. Many of the newly developed antimicrobial substances studied so far are difficult to apply in vivo due to their poor solubility, cytotoxicity to host tissues and their short half-life, as well as their poor permeability across biological membranes [8,9]. Therefore, the newly developed antimicrobial agents, including dendrimers, should overcome the above-mentioned disadvantages.

After the synthesis of the first polypropyleneimine (PPI) dendrimers, a large number of dendrimeric structures were synthesized, with application in nano- and biotechnology, catalysis, medicine, pharmacy, etc. [10,11,12,13,14,15,16]. Some of the most commonly studied classes of dendrimers are PPI and polyamidoamine (PAMAM) and their derivatives.

Dendrimers have a well-defined structure composed of multiple reaction centers that originate from a core with gradual symmetrical branching in each subsequent layer, resulting in different generations of dendrimers. After the third generation of dendrimers, internal cavities are formed, which give them their characteristic spherical shape, as well as a multivalent surface that can interact with a large number of biologically active substances. Dendrimers contain a large number of identical or different end functional groups, which gives great opportunities for their purposeful modification by adding ions, drugs, enzymes, or other biomolecules and change in their properties [17,18,19,20,21,22]. The size, composition, charge, topology, and viscosity of dendrimers can be tightly controlled during their synthesis, giving great opportunities for targeted drug delivery systems for enzymes, contrast agents, genes, antibacterial, antiviral and anti-amyloid drugs [23,24,25,26,27]. Nowadays, they are progressively used in medicine and pharmacy, e.g., in imaging diagnostics, in disease therapy, as biosensors, in immunotherapy, for therapy of cancer, etc. [28,29,30,31,32,33].

Over the last years, dendrimers and their modified derivatives have gained an increasing interest in research as an alternative to traditional widely used antimicrobial medicinal substances [34,35,36,37]. Due to their unique structure and properties, dendrimeric compounds have the following advantages: nanoscale 1–100 nm, which facilitates their entry into cells; combination of the properties of low- and high-molecular substances; good water solubility and increased permeability; and low polydisperse index, which allows the transfer of drugs by analogy with liposomes, cubosomes, etc. [38,39,40,41].

The aim of the present work was to study the molecular mechanisms of interaction between model phospholipid membranes (monolayers and liposomes) composed of 1-palmitoyl-2-oleoylphosphatidylcholine (POPC) with two recently synthesized by peripheral modification of a first-generation PPI with 1,8-naphthalimides dendrimers with proven antibacterial activity. By using complementary and highly informative biophysical methods and models, namely Langmuir monolayers, large unilamellar vesicles (LUVs), giant unilamellar vesicles (GUVs), and multilamellar vesicles (MLVs), new fundamental knowledge will be acquired to reveal the mechanism of action of newly synthesized dendrimers on model membranes. The results obtained will contribute to the antimicrobial potential of dendrimers with a focus on their application in pharmacology and clinical practice.

## 2. Materials and Methods

### 2.1. Materials

The lipid POPC (1-palmitoyl-2-oleoyl-sn-glycero-3-phosphocholine) was purchased from Avanti Polar Lipids (Alabaster, AL 35007, USA). Dimethyl sulfoxide (DMSO) for biological investigation was used as obtained from Sigma-Aldrich (Darmstadt, Germany). Methanol, chloroform, and diethyl ether as organic solvents and the fluorescence probe Laurdan (2-(dimethylamino)-6-dodecanoylnaphthalene) of spectroscopic grade were used as obtained from Sigma-Aldrich (Germany).

### 2.2. Synthesis of Dendrimers with Antibacterial Activities

**Figure 1:**
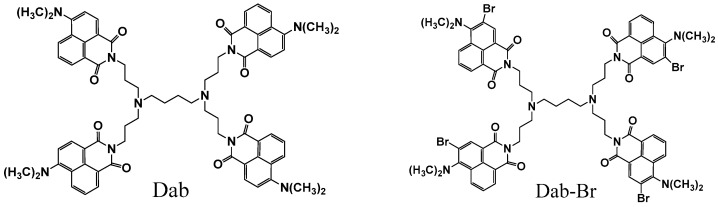
Chemical structure of Dab and Dab-Br.

The synthesis and characterization of modified with 4-*N*,*N*-dimethylamino-1,8-naphthalimide (diaminobutyl residue in the core, or Dab) and 3-bromo-4-*N*,*N*-dimethylamino-1,8-naphthalimide (Dab-Br) poly(propylene imine) dendrimers (Figure 1) was described [42].

### 2.3. Langmuir Monolayer Studies

All measurements were performed by the Wilhelmy method using Langmuir trough Micro-Trough X (Kibron Inc., Helsinki, Finland) equipped with two movable barriers, Teflon MicroTrough G1 (L × W × D = 260 mm × 80 mm × 5 mm), and a standard Kibron plate attached to the microbalance sensor head. POPC was dissolved in chloroform to a concentration of 1 mg/mL. Dendrimers were dissolved in DMSO to concentration of 0.1 mg/mL.

The POPC solution was spread in small portions with a Hamilton microsyringe (Hamilton Central Europe S.R.L., Timis County, Romania) over a subphase of deionized water (pH = 5.5; the pH was not adjusted using any reagents). The formed lipid monolayer was left for 15 min to ensure chloroform evaporation and monolayer equilibration. Then, the dendrimer solution was spread in small portions with a Hamilton microsyringe at the surface of the monolayer, and the film was left for 15 min to obtain a uniform surface mixture. The quantity of the dendrimer solution was calculated to a ratio of POPC/dendrimer = 50:1 and 25:1 (in terms of number of molecules). After reaching an equilibrium surface pressure (π, mN/m), the mixed monolayers were compressed at slow rate (20 mm/min), and the π-area (A, Å^2^/molecule) isotherms were recorded. The surface pressure was measured with an accuracy of ±0.1 mN/m. All experiments were conducted at room temperature (22 ± 1 °C). Each measurement was repeated at least three times.

The compressional modulus (Cs^−1^, mN/m) was obtained from the π-A isotherms according to the following equation:

$${Cs}^{-1}=-A_{\pi }{\left(\frac{d\pi }{\mathrm{dA}}\right)}_{T},$$

where A_π_ is the area per molecule at the indicated surface pressure π.

The analysis of the data obtained was performed using OriginPro 9.0 software.

### 2.4. FTIR-ATR Spectroscopy of MLVs

POPC MLVs were prepared in bi-distilled water according to the mechanical dispersion method [43,44,45]. POPC solutions were prepared with chloroform at a concentration of 1 mg/mL. Bi-distilled water was filtered through 0.2 μm sterile syringe filters (Corning Incorporated, Wiesbaden, Germany) prior to use. Glass vials were used for the deposition of solvent-free dry lipid films after chloroform evaporation under vacuum for at least 4 h. Afterwards, the lipid films were hydrated with bi-distilled water, an 80 µg/mL DMSO aqueous suspension, or a dendrimer–DMSO aqueous suspension and subsequently diluted to achieve s final concentration of the dendrimers of 5, 40, and 80 µg/mL. The samples containing dendrimers were obtained from 3 mg/mL stock solutions in DMSO. Each hydrated lipid sample was vortexed and subsequently placed in an ultrasonic bath.

A spectrophotometer Vertex 70 (Bruker, Rosenheim, Germany) was used in geometry of attenuated total reflectance (ATR) for the acquisition of Fourier-transform infrared (FTIR) spectra of aqueous suspensions of POPC MLVs. The standard PIKE MIRacle ATR accessory was equipped with a ZnSe reflection prism yielding three internal reflections and covered by a diamond plate. The low frequencies of the scanned wavenumber range 4000–600 cm^−1^ scanned were limited by the transmission edge-cut of the ZnSe crystal. MLV samples were filled into an O-ring pool (diameter 8 mm, torr−1 mm) placed on the diamond plate. Each spectrum was obtained by averaging 100 interferograms with resolution of 2 cm^−1^. All measurements were performed at 22 ± 1 °C.

### 2.5. Laurdan Fluorescence Spectroscopy of LUVs

LUVs formation was performed as follows: an aliquot of stock solution of 10 mg/mL POPC dissolved in chloroform was evaporated from the organic solvent by a stream of nitrogen. The formed lipid film was placed under a vacuum overnight to completely remove the organic solvent traces. Then, for POPC liposome formation, hydration with distilled water (pH 5.5; pH was not adjusted using any reagents) of the dried lipid film to achieve concentration of 1 mM POPC was performed. After that, vortex and sonication were carried out to obtain multilamellar vesicles. This procedure was repeated three times. For LUVs formation, an extrusion with LiposoFast-Basic extruder (Avestin, Ottawa, Canada) containing polycarbonate filters with a diameter of pores 800 nm (11 passages) and 100 nm (21 passages) was carried out. The newly created LUVs of POPC before and after treatment with different concentrations of Dab and Dab-Br dendrimers dissolved in DMSO, with pure DMSO as control, were used to determine the membrane lipid order by Laurdan fluorescence spectroscopy. To obtain the desired final dendrimer concentrations (5, 40, and 80 µg/mL) in 1 mL lipid suspension in the cuvette, corresponding amounts of 1 mg/mL dendrimer stock solution were added. All experiments were conducted at room temperature (22 ± 1 °C).

Laurdan spectroscopy was applied using the fluorescence environment-sensitive probe Laurdan localized in the membrane bilayer at the level of the glycerol backbone of the phospholipid to investigate the degree of membrane lipid order [46]. An aliquot of Laurdan, solubilized in methanol, was added to the lipid dissolved in chloroform at a 1:200 Laurdan/lipid molar ratio. When Laurdan is partitioned into the membrane bilayer, the changes in water content in the membrane induce alteration in the probe’s excitation and emission spectra. It can be quantified by calculating the generalized polarization (GP) using fixed emission wavelengths according to the following equation:

$${\mathrm{GP}}_{\mathrm{ex}}=\frac{\left(I_{440}-I_{490}\right)}{\left(I_{440}+I_{490}\right)},$$

where I_440_ and I_490_ are the emission intensities at a characteristic wavelength of the gel/liquid-ordered (L_o_) phase (440 nm) and the liquid-disordered (L_d_) phase (490 nm). Samples were excited at 355 nm. The emission spectra of Laurdan were recorded from 390 to 600 nm. At GP values (−1), the membrane is defined as L_d_ phase state and with values (+1)—as gel/L_o_ phase state. The final lipid concentration in the quartz cuvette was 0.2 mM. All measurements were performed at room temperature (22 ± 1 °C) using a Hitachi spectrofluorometer. The analysis of the obtained data was performed using OriginPro 9.0 software.

### 2.6. Zeta Potential and Size of LUVs

The ζ potential and the size of the pure (control) and dendrimers POPC-treated vesicles were measured by dynamic light scattering (DLS) at room temperature (22 ± 1 °C). Zeta sizer Advance Series Instrument (Malvern Analytical, Malvern, UK), supplied with a 4 mW HeNe laser (632.8 nm) and angle detection of 173°, was used to determine POPC vesicle size. Zeta potential was measured by the same analyzer using the principle of electrophoresis. The samples of 0.2 mM control and dendrimer-treated LUVs dissolved in distilled water were placed in a specific U-shape capillary cuvette, and an external electric field was applied. The velocity or electrophoretic mobility (u) under the influence of an electric field was measured by laser Doppler velocimetry. The ζ potential was calculated using the Helmholtz–Smoluchowski equation:

$$\zeta =\frac{\eta u}{\varepsilon_{r}\varepsilon_{0}},$$

where η is the viscosity, ε_r_ the dielectric constant of the aqueous phase, and ε_0_ the dielectric permittivity under vacuum [47].

Six measurements per sample were performed to calculate the standard deviation of the obtained values. All the data were analyzed using OriginPro 9.0 software.

### 2.7. GUVs Electroformation and Video Microscopy

The electroformation is an extensively used method for giant unilamellar vesicle preparation [48]. A one-component POPC solution at concentration of 0.5 mg/mL was prepared in mixture of diethyl ether/methanol/chloroform in a ratio of 70/10/20 *v*/*v*/*v*. GUV electroformation involves the following steps: 1 μL lipid solution is deposited in 3 places on each platinum electrode. Then, the solvent was evaporated under a vacuum for at least 45 min. Before the hydration of the dried lipid film with distilled water, 10 Hz alternating current (AC) and 100 mV peak-to-peak were applied to the electrodes. The AC amplitude was raised gradually over one hour up to about 500 mV. At least 20 GUVs with 30–90 μm sizes were observed within three hours. The vesicle treatment with dendrimers was carried out by adding aliquots of dendrimer–DMSO solution to the water surface away from the electrodes where the vesicles were formed. All experiments were conducted at room temperature (22 ± 1 °C).

GUVs were observed by Zeiss Axiovert 135 microscope equipped with 40× long working distance objective lens. Observations were recorded using Zeiss AxioCam HSm CCD camera connected to an image recording and processing system (Axiovision, Carl Zeiss, Wien, Austria).

## 3. Results and Discussion

### 3.1. Langmuir Monolayer Studies of POPC Treated with Dendrimers

Langmuir’s monolayer technique is widely used to study the interaction between different membrane active substances and biomembranes, including the mechanism of the toxicity of different antimicrobial agents on different pathogens [49,50,51]. The advantages of this model are that membrane characteristics such as orientation and packaging and lipid composition as well as experimental conditions like temperature, ionic strength, and subphase pH can be controlled [52].

To evaluate the molecular mechanism of action of Dab and Dab-Br on biomembranes, we studied the effects of the respective compounds on POPC monolayers by Langmuir monolayer technique. This lipid, although less abundant in bacterial membranes [53], represents the human cell membrane since it is one of the most common phospholipid types extensively used as a mimic for eukaryotic membranes [54]. Our conception to use POPC as a model lipid was to examine if there would be any deterioration effect of the dendrimers on eukaryotic cells in addition to their destructive activity on bacterial membranes, considering their eventual application as antimicrobial cotton fabrics or as drug delivery systems in humans.

**Figure 2:**
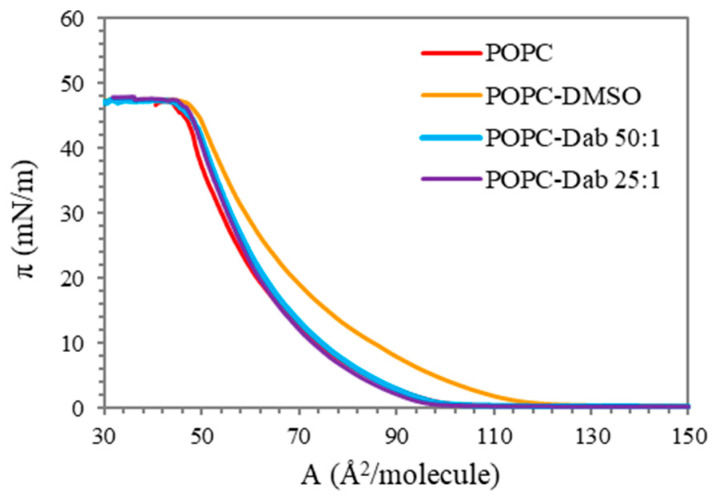
Surface pressure (π, mN/m) vs. molecular area (A, Å^2^/molecule) isotherms of POPC monolayers before and after the addition of Dab.

The surface pressure versus area isotherms recorded upon compression of pure lipid and after the addition of Dab are shown in Figure 2. The mixture of POPC and the solvent of the dendrimer, DMSO, is also shown to distinguish its effect on the monolayers. The pure lipid isotherm is in good agreement with those published elsewhere [55,56]. It follows the characteristic behavior of POPC monolayer with a lift-off at about 100 Å^2^/molecule. Upon compression, POPC remained in liquid-expanded phase without any visible transition until it reached a collapse surface pressure of approximately 47 mN/m. The addition of Dab to the lipid monolayer, at first sight, did not change the isotherm shape regardless of the dendrimer concentration. However, we found a slight effect of the DMSO–lipid mixture as compared to the pure lipid: the lift-off started at a higher area per molecule, and the curve was shifted to the right, suggesting that DMSO itself inserts into POPC monolayer and consequently interrupts the packing of the lipid molecules. This result confirmed the observation obtained by Laurdan fluorescence spectroscopy of POPC liposomes (see below). The effect of DMSO on the pure POPC monolayer was expected since it was found by other authors that, depending on its concentration, DMSO induces changes in membrane fluidity and integrity [57,58,59,60,61].

Considering that the dendrimers studied were dissolved in DMSO, the lack of any visible difference between the isotherms of POPC and POPC-Dab (in both concentrations studied) actually misleads one to believe that the dendrimer had no effect on the lipid monolayer. In fact, when we compare POPC-DMSO and POPC-Dab isotherms (Figure 2) we can speculate that Dab itself compensates for the effect of DMSO or competes with it for the area between the lipid molecules. This eventually results in interaction with the lipid molecules increasing the molecular arrangement of POPC monolayer, as seen also by our experiments with liposomes as a model membrane. Similar studies using the saturated dipalmitoyl phosphatidylcholine (DPPC) monolayer as a model membrane and G2 PAMAM dendrimers, single-walled carbon nanotubes (SWCNT) and dendrimer-SWCNT conjugates (G2, G4, and G6) were performed by Cancino et al. [62]. The authors reported that the addition of G4-SWCNT and G6-SWCNT to the monolayers had no noticeable effect on π-A isotherms of DPPC monolayers except for a small change in collapse pressure. They concluded that these two conjugates did not change the lipid packing but adsorbed at the interface. However, PAMAM-G2, SWCNTs, and G2-SWCNT conjugates led to an incorporation into the monolayer. Wilde et al. [63] demonstrated a concentration-dependent incorporation of carboxylate- (G4.5) and amine- (G5) terminated dendrimers into DPPC monolayers: negligible or no dendrimer penetration into the monolayer was observed at low dendrimer concentration, but higher dendrimer concentrations led to a more pronounced effect.

**Figure 3:**
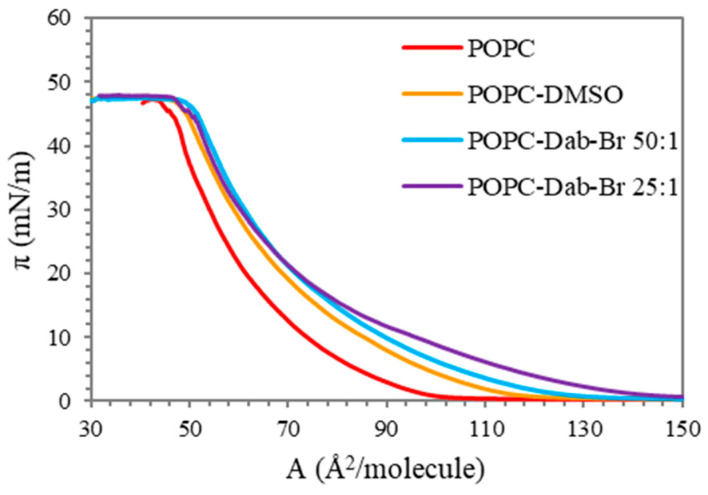
Surface pressure (π, mN/m) vs. molecular area (A, Å^2^/molecule) isotherms of POPC monolayers before and after the addition of Dab-Br.

The increase in the positive charge of the brominated dendrimer affected the POPC monolayer by a different manner as compared to Dab (Figure 3). The comparison between the pressure-area isotherms of the pure lipid and those obtained after the addition of Dab-Br shows that the brominated dendrimer had no significant effect on POPC film as compared to DMSO at a surface pressure relevant to the biological membranes (25–30 mN/m). The lift-off of the three isotherms (POPC-DMSO and POPC-Dab-Br at ratios of 50:1 and 25:1) was at a greater area per lipid molecule. This tendency was observed throughout the compression. However, at low surface pressure, Dab-Br led to a more significant deviation from the pure lipid behavior as compared to dimethyl sulfoxide, and the effect was concentration-dependent. After reaching surface pressure values of approximately 20 mN/m, the influence of Dab-Br on POPC film as compared to DMSO was reduced, most probably because of the competition between the molecules of the solvent and the dendrimer for the decreased free area between the lipid molecules in the monolayer. Based on the obtained results, we can conclude that Dab-Br inserts into the POPC monolayer, changing its structure. Moreover, the mixed isotherms followed the shape of POPC, which shows a stable interaction between the dendrimer and the lipid.

The effects of Dab and Dab-Br on the lipid monolayer could be explained by the assumption that the interaction between the dendrimers and POPC at the region of the lipid head group most probably is due to electrostatic interaction. However, Dab is likely to penetrate the unsaturated fatty acyl chains of POPC, leading to an alignment of the lipid tails, whereas the bigger and more positively charged molecule of Dab-Br inserts mainly into the lipid head group region. This assumption was further confirmed by FTIR-ATR spectroscopy of POPC and POPC-dendrimers MLVs.

The compressibility of the monolayers or the lateral elasticity given by the compressional modulus (Cs^−1^) provides an additional assessment of the physical properties of the Langmuir monolayer based on pressure-area isotherms [64,65]. Higher values of the maximum compressional modulus correspond to a decreased monolayer elasticity [52]. The compressibility gives valuable information about the lateral packing of the molecules and the phase transition of the film during the compression and the effect of different surface active compounds on the monolayer properties [66,67]. The presence of more than one peak on the graph indicates a phase transition occurring in the system [68].

**Figure 4:**
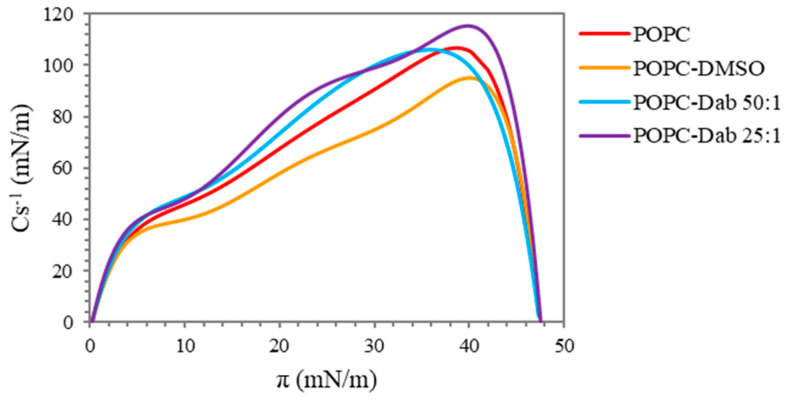
Compressional modulus (Cs^−1^, mN/m) versus surface pressure (π, mN/m) of POPC monolayers before and after the addition of Dab.

The compressional modulus versus surface pressure of the pure POPC and after the addition of Dab is presented in Figure 4. The compressibility of the pure lipid had the characteristic pattern for POPC with one pronounced peak, which is in agreement with those reported by other authors [55,69]. The maximum value of Cs^−1^ reached about 105 mN/m. Based on the literature, values of the compressional modulus between 100 and 250 mN/m are indicative of the liquid-condensed phase at high surface pressure [70,71]. The addition of the solvent, DMSO, to the lipid monolayer led to a noticeable decrease in Cs^−1^ maximum to 95 mN/m, i.e., it exhibits a fluidizing effect and the appearance of two asymmetric peaks. These observations suggest a disordering effect of the solvent on the monolayer structure with the coexistence of liquid-expanded and liquid-condensed phases. The addition of Dab compensated for the effect of DMSO on the lipid monolayer: the lower concentration of the dendrimer (POPC/Dab 50:1) reached the same maximum value of Cs^−1^ as the pure lipid and had the same graph pattern. In addition, the peak was shifted to slightly lower values of surface pressure, suggesting that the most condensed regions of the mixed lipid–dendrimer monolayer occurred in a slightly bigger molecular area. A similar conclusion was proposed by Cancino et al. [62] when G2 PAMAM, SWCNTs, and G2-SWCNT conjugates were added to the DPPC monolayer. A bigger ordering effect, i.e., increased rigidity of the mixed monolayer, was noticed with the higher Dab concentration applied (POPC/Dab 25:1), especially considering that it was dissolved in DMSO, which produced an opposite effect. Although the Cs^−1^ maximum was at the same surface pressure, the visible presence of two peaks was observed, indicating a phase transition. Most probably, Dab was incorporated into POPC monolayer at the region of the unsaturated oleoyl chains, which led to straightening of the lipid tails.

**Figure 5:**
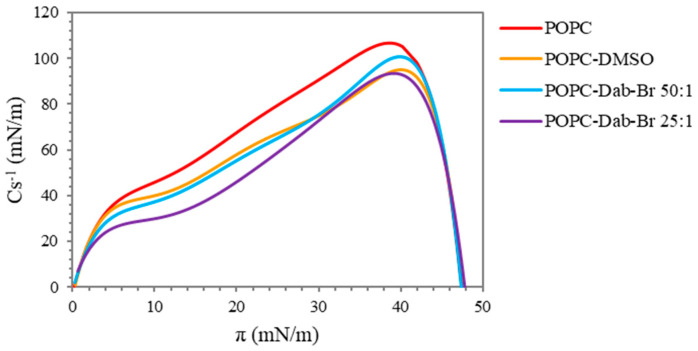
Compressional modulus (Cs^−1^, mN/m) versus surface pressure (π, mN/m) of POPC monolayers before and after the addition of Dab-Br.

The brominated form of the dendrimer, Dab-Br, led to a decrease in the compressional modulus at both concentrations studied (Figure 5). Although the lower concentration of the dendrimer (POPC/Dab-Br 50:1) partially compensated for the effect of DMSO at high surface pressure (i.e., condensed POPC monolayer), indicated by the higher values of Cs^−1^ as compared to POPC-DMSO, the higher concentration of applied Dab-Br led to the same effect as DMSO. However, up to π values of approximately 30 mN/m, which is the physiologically relevant surface pressure, the compressional modulus values remained lower as compared to POPC-DMSO and to the pure lipid. The obtained results indicate an increase in the elasticity of the POPC monolayer with the appearance of liquid-expanded phase in the film. Further compression of the mixed monolayers showed similar effects of Dab-Br and the solvent DMSO. These findings suggest that Dab-Br incorporates between the molecules of the lipid, predominantly in the region of the head groups in the presence of free area between POPC molecules. Most probably, the penetration of the brominated dendrimer is due to electrostatic interactions between the positive charge at the tertiary nitrogen atoms from the dendrimer core and the negatively charged part of phosphatidylcholine.

The Langmuir monolayer studies on the effect of Dab and Dab-Br on a model membrane composed of POPC revealed that the two types of dendrimers interact with the monolayer by applying a different strategy. Based on π-A isotherms and the compressibility of the monolayers, we suggest that Dab incorporates in the kink formed by POPC unsaturated tails, leading to molecular ordering in the plane of the monolayer. On the other hand, due to its higher positive charge, Dab-Br interacts electrostatically with the negatively charged phosphate in phosphatidylcholine. Our findings are in agreement with the studies of Tian et al. [72], who used combined Langmuir monolayer experiments and coarse-grained molecular dynamics simulations. The authors demonstrated that PAMAM dendrimers (G3-OH and G5-OH) had little effect on the surface pressure-area isotherm profiles of DPPC monolayer. The addition of positively charged surface terminals (-NH_2_) significantly increased the penetration of both G3 and G5 PAMAM dendrimers into the monolayer due to electrostatic interactions with the lipid head group. According to Tian and co-workers, charged PAMAM dendrimers directly interact with the lipid phosphate head groups, while charge-neutral PAMAM dendrimers embedded into the hydrophobic region of the monolayer.

### 3.2. FTIR-ATR Spectroscopy of POPC Liposomes Containing Dab and Dab-Br Dendrimers

Infrared spectroscopy provides insight into changes in the structure and mobility of individual moieties of lipids. FTIR is a powerful tool in absorption spectroscopy, yielding valuable information about the vibrational modes of molecular bonds and combining non-destructive analysis and rapid data collection with high sensitivity and resolution. The ATR technique significantly augments the sensitivity of the acquisition, thereby reducing the required sample thickness for transmittance measurements.

FTIR-ATR experiments were conducted in order to detect the effect of dendrimers on molecular organization of POPC bilayers as a function of Dab and Dab-Br concentration in the bulk phase [73,74,75]. The spectral distance between the vibrational bands of lipid molecules’ moieties rendered possible the relatively selective observation of the hydrophobic chain dynamics and the induced perturbations of the polar hydrophilic heads [76,77,78]. The spectral acquisition was carried out in situ with aqueous suspensions of vesicles. The strong absorption bands of the bulk water were removed from the spectra to provide a clear picture of the above bands by keeping the role of the lipid hydration in the suspension on the studied interactions as previously discussed [79,80,81].

Several distinct spectral regions can be assigned to the specific vibrations in the different moieties of POPC molecules [82,83]. The spectra at the highest frequencies 2800–3000 cm^−1^ characterize the C-H symmetric and asymmetric stretching vibrations in the hydrophobic chains, to which the -CH_3_ modes of the chain ends and the choline groups can also contribute. The frequencies 800–1300 cm^−1^ are characteristic of the vibrations in the headgroups at the membrane interface and of the rocking modes in the -CH_2_ chains.

**Figure 6:**
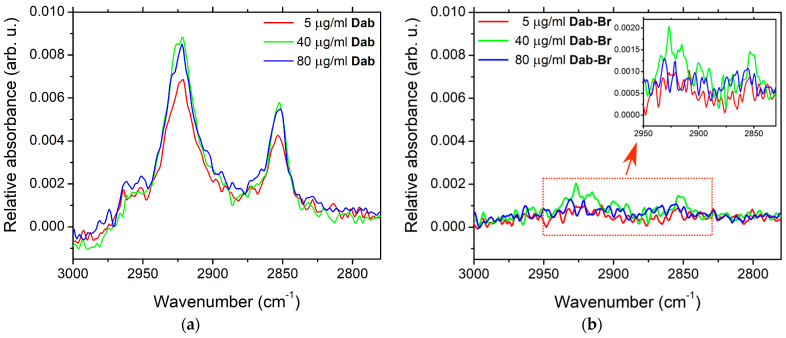
FTIR-ATR spectra of POPC MLV suspensions in the zone of the C-H stretching vibrations for (**a**) Dab; (**b**) Dab-Br. For comparison, both panels are identically scaled; the inset shows the same spectra with Y axis rescaled to better visualize the C-H stretching vibrations in -CH_2_ and -CH_3_ groups.

The measured spectra are presented and discussed by spectral regions as introduced above. Due to the strong interactions of DMSO with the lipid bilayers, we performed a series of control measurements of MLV suspensions containing only DMSO with a concentration corresponding to its quantity in the Dab and Dab-Br samples studied. The effect on the lipid vibrational modes in the membrane for each studied dendrimer concentration was discussed after subtracting the spectra of the relevant dendrimer-free control sample. The lack of vibrational modes in this relative absorbance spectrum correlate with the absence of effect of the corresponding dendrimer compared to the control spectrum of POPC-DMSO samples. The FTIR-ATR spectra of POPC MLV suspensions containing 5, 40, or 80 µg/mL of Dab or Dab-Br dendrimers are presented in Figure 6. The most distinguished and relatively well-established bands are those of the C-H stretching vibrations in -CH_2_ and -CH_3_ groups of the hydrocarbon chains of POPC-Dab MLVs (Figure 6a). As the lipid membrane has a densely packed structure, external perturbations can cause a detectable response, thus providing information about the dynamics of the hydrophobic tails. The peak positions remained unchanged for all samples, while their bandwidths decreased with the addition of Dab, which indicates the dendrimer’s confining effect on the lipid tail mobility [84,85]. Only a slight effect of Dab-Br on these modes was noticed, indicating the negligible alteration of the lipid backbone by the brominated dendrimer (Figure 6b).

**Figure 7:**
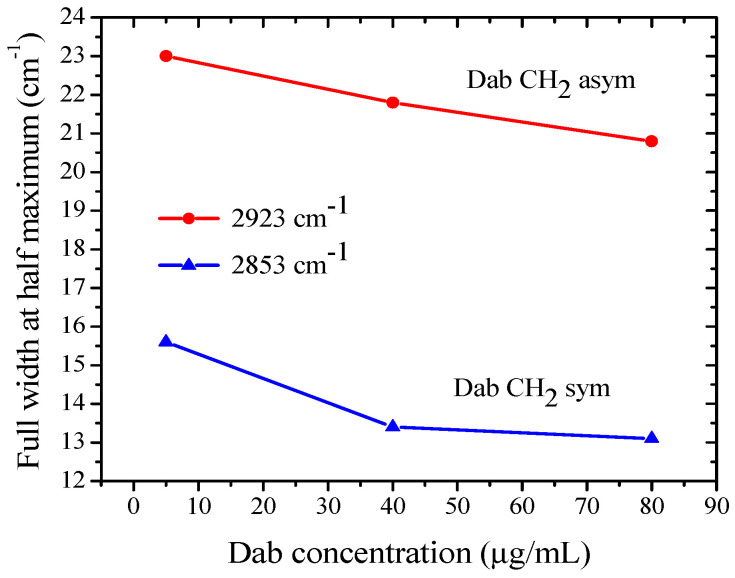
The band width at half maximum of -CH_2_ stretching vibrations as a function of Dab dendrimer concentration in the suspension.

The bands of the -CH_2_ symmetric and asymmetric stretching vibrations for all non-brominated Dab samples were registered at 2853 cm^−1^ and 2923 cm^−1^, respectively (Figure 6a). The band width at half maximum of -CH_2_ stretching vibrations at these frequencies as a function of Dab dendrimer concentration in the suspension is presented in Figure 7. With increasing Dab concentration in the suspension, a slight width reduction for both symmetric and asymmetric bands can be observed. As the dipole moment of the -CH_2_ stretching vibrations is in the C-C-chain plane [82], we can assume a “hardening” effect of Dab, leading to the obtained increase in the band intensity.

The bands of -CH_2_ asymmetric vibrations in all spectra are structurally complicated at different degrees by the rise of the -CH_3_ asymmetric modes at 2953 cm^−1^ as a shoulder. The deconvolution of the structured bands revealed that -CH_3_ sub-bands slightly decreased in width, and their intensity increased with the concentration of Dab. The activation of these vibrations is probably caused mainly by the end -CH_3_ groups of dendrimers; however, the excitement of -CH_3_ in choline moieties and hydrocarbon tails of lipid can also contribute.

**Figure 8:**
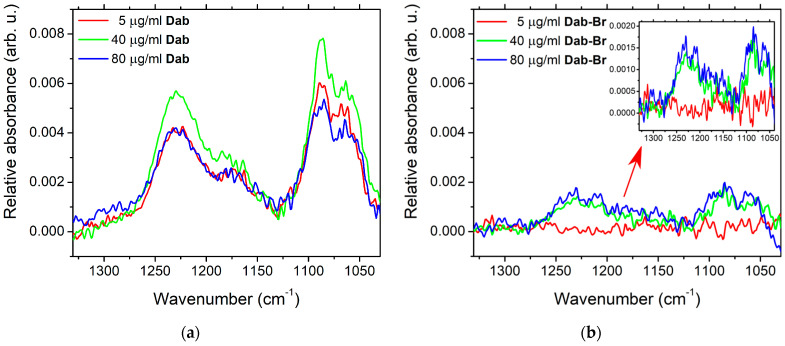
FTIR-ATR spectra of POPC MLV suspensions in the zone of vibrations of the phosphate groups for (**a**) Dab; (**b**) Dab-Br. For comparison, both panels are identically scaled; the inset shows the same spectra with Y axis rescaled to outline the bands in the presence of the brominated dendrimer.

The effect of POPC–dendrimer interaction in the range of the active vibrations in the lipid headgroup is presented in Figure 8 as a broad band from 1270 to 1120 cm^−1^ originating from the vibrations of the phosphate moiety. The two sets of spectra display the differences in the phosphate groups’ vibrations upon the influence of the two types of dendrimers. More pronounced lipid–dendrimer polar interactions of Dab (Panel a) are reported compared to Dab-Br (Panel b). The two strong bands around 1250 and 1085 cm^−1^ are characteristic for the antisymmetric and symmetric stretch of the double-bond P=O, respectively. The changes in the band height and width correspond to alterations in the mobility of the lipid polar headgroups in the presence of dendrimers [76]. This effect is weaker for the brominated samples, as shown in Figure 8b.

FTIR-ATR measurements clarify the effect of the studied dendrimer species on lipid molecular organization in the membrane. The reported spectral alterations support the conclusion that Dab changes the aliphatic chain order and promotes the hydrocarbon tails packing in the bilayer. On the other hand, the presence of the brominated dendrimer has no effect on the characteristic vibrational modes for POPC hydrocarbon tails compared to the control samples. Both dendrimer molecules perturb the lipid headgroup mobility. This effect is more pronounced for Dab in relation to its deeper impact on the membrane hydrocarbon region. Conversely, the interactions of the brominated molecule with lipids appear to take place at the polar surface of the membrane, as concluded from the spectral characteristics of the different POPC moieties’ vibrations.

### 3.3. Membrane Lipid Order of LUVs Treated with Dendrimers

Vesicles are crucial model membranes in biological and biophysical research due to their structural complexity and physiological relevance. They offer distinct advantages over monolayers for studying membrane-related phenomena [86,87,88]. Vesicles mimic the bilayer architecture of cellular membranes, enabling investigation of fundamental membrane properties like lipid organization, fluidity, and permeability [89,90]. This facilitates studying membrane-associated processes, such as interactions of lipids with biologically active molecules and nanoparticles like dendrimers, membrane adsorption and fusion, and transmembrane transport, providing insights into cellular functions and pathology [91,92,93]. Researchers can tailor vesicle properties to mimic specific biological membranes or to investigate the effects of lipid composition on membrane structure and functions. Moreover, vesicles exhibit enhanced stability compared to monolayers due to their enclosed structure [94,95]. This stability minimizes artifacts from external perturbations and enables long-term studies of membrane dynamics and functions under physiologically relevant conditions. The controlled size distribution of vesicles allows for precise control of the membrane composition, curvature, and surface area. Thus, the effects of membrane structure and geometry on membrane-associated processes like dendrimer binding, lipid ordering and sorting, and subsequent membrane deformation could be investigated [96,97].

**Figure 9:**
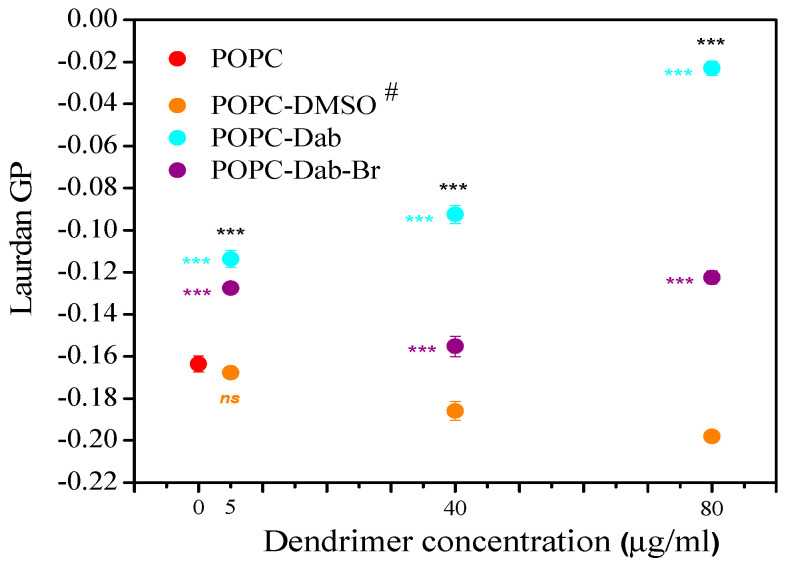
Laurdan GP values of POPC vesicles versus the concentration of Dab and Dab-Br (µg/mL). # designates DMSO volumes (µL) corresponding to those of POPC-dendrimer suspensions: 0, 5, 40, and 80 µL DMSO. Error bars represent the SD of three independent experiments. The data are analyzed with one-way ANOVA; *** denotes *p* < 0.001 (colored stars compare POPC-dendrimers including DMSO, whereas dark ones compare pure POPC-dendrimers); ns: not significant data.

Our study demonstrates that the addition of dendrimers results in significant differences in the lipid order of POPC vesicles (Figure 9).

DMSO alone was also added to the lipid suspension to distinguish the effect of the carrier from that of the dendrimer one. We found a slight effect of DMSO on the POPC vesicles, expressed in a decreased membrane lipid order. As the DMSO amount was increased, the lipid order was reduced. This result is in agreement with those published in the literature [57,58,59,98,99]. It was found that DMSO at low concentrations induced membrane fluidization and thinning. By using molecular simulations, it was shown that high DMSO concentrations could induce pore formation into the membrane and lipid desorption from the membrane, causing the lipid bilayer breakdown at high DMSO concentrations [58].

**Figure 10:**
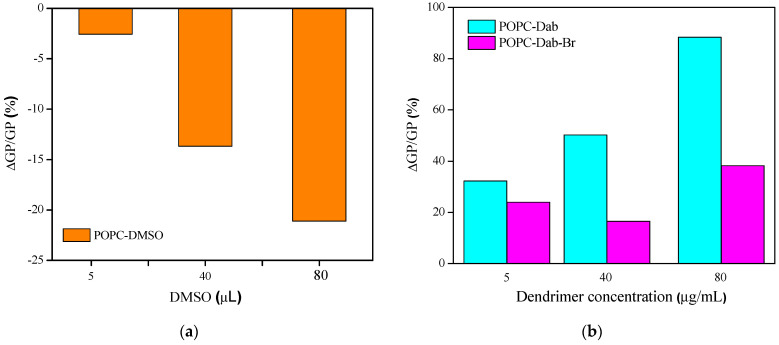
The relative change of GP (∆GP/GP, %) as a function of (**a**) DMSO (µL) and (**b**) dendrimer concentration (µg/mL) for POPC LUVs.

Dendrimers dissolved in DMSO also affected the membrane organization but in the opposite way. The lowest concentration (5 µg/mL) of both dendrimers increased the degree of lipid order in the bilayer. Vesicles treated with 40 and 80 µg/mL Dab demonstrated the same tendency to increase the lipid order parameter. An identical trend was observed only at the highest concentration for Dab-Br. The relative GP change was calculated to assess the DMSO and dendrimers’ effects on the lipid order (Figure 10). Relative changes of GP (∆GP/GP_0_, %) were calculated for DMSO (Figure 10a) and dendrimer treatment (Figure 10b), where ∆GP_DMSO or dendrimer_ = GP_DMSO or dendrimer_ − GP_0_. GP_DMSO_ indicates the GP value of POPC vesicles treated with DMSO, while GP_0_ corresponds to pure POPC vesicles (Figure 10a). Similarly, ∆GP_dendrimer_ = GP_dendrimer_ − GP_0_ was calculated, where GP_dendrimer_ indicates the GP value of POPC vesicles treated with dendrimer, while GP_0_ corresponds to POPC treated with DMSO (Figure 10b). The positive values of ∆GP/GP indicate the lipid ordering effect induced by dendrimers, while the negative values correspond to fluidizing the POPC bilayer. Our results demonstrated that the effects of DMSO and dendrimers on lipid order were opposite. DMSO alone (from 0.005 to 0.08 volume %) reduced the POPC membrane order to about 20%. Dab increased the lipid order by 90%, whereas Dab-Br had only 30% with the highest studied dendrimer concentration (80 µg/mL).

### 3.4. LUVs Zeta (ζ) Potential Changes and Size Distribution Under Dendrimer Treatment

**Figure 11:**
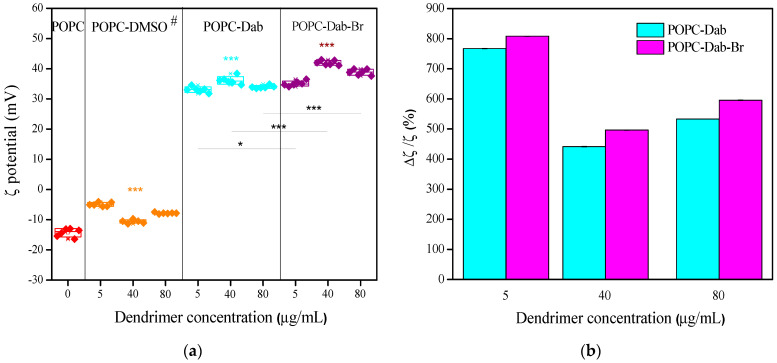
(**a**) Zeta (ζ) potential (mV, panel a) and (**b**) relative ζ potential (∆ζ/ζ, %, panel b) of POPC vesicles as a function of Dab and Dab-Br concentration (µg/mL). # designates DMSO volumes in µL corresponding to those of POPC-dendrimer suspensions: 0, 5, 40, and 80 µL DMSO. Error bars represent the SD of two independent experiments with 6 repeats for each sample. The data are analyzed with one-way ANOVA; *** and * denote *p* < 0.001 and *p* < 0.05 (colored stars compare POPC-dendrimers including DMSO, whereas dark ones compare pure POPC-dendrimers).

In addition to the effect of the dendrimers on POPC liposomes’ molecular organization, the measurement of ζ potential showed that Dab and Dab-Br also affected the electrical properties of the lipid bilayer. The negative values of ζ potential for POPC LUVs in distilled water were measured to be about −15 mV (Figure 11a). A negligible effect of DMSO on POPC membranes was detected, expressed in a slight increase in ζ potential values. The interaction between the dendrimers and POPC clearly showed a significant increase in ζ potential (reaching positive values) with increasing dendrimer concentrations. POPC vesicles treated with only 5 µg/mL Dab or Dab-Br were enough to change the ζ potential of POPC membranes from negative −5 mV to positive values +35 mV. The increasing dendrimer concentrations slightly enhanced this parameter. The ζ potential values of each dendrimer in water were found to be comparable to those of POPC LUVs with dendrimer at the studied concentrations. The relative change in ζ potential (∆ζ/ζ potential, %, analogously to the relative change in GP) ranged from 400% to 800%, indicating strong interactions between dendrimers and POPC molecules as well as significant modifications in the surface characteristics of the studied lipid membrane (Figure 11b). Furthermore, ζ potential values of POPC membranes treated with Dab-Br were about 10% higher than those with Dab. The observed positive values of ζ potential of POPC LUVs in the presence of cationic dendrimers in our study are in agreement with those obtained for other types of dendrimers, depending on their size and chemical modification [100,101,102,103]. The positively charged dendrimers interact strongly with the negatively charged phosphoryl groups in the lipid headgroups. This electrostatic interaction can lead to clustering of lipids around the dendrimers, altering the local lipid packing and order. Such interactions apparently result in decreased membrane fluidity and increased rigidity in the vicinity of the dendrimer, as the lipid molecules are drawn closer together by the dendrimer’s presence. Indeed, by using NMR, it was shown that the molecular order parameter values increased upon adding dendrimer (G7 or G5) to the DMPC vesicles, indicating that the dendrimer binding reduces the flexibility of the fatty acyl chains. Furthermore, the effect varied with dendrimer size, as the G5 dendrimer had a greater impact on the dynamics of the hydrophobic core [104]. Larger dendrimers exhibited a reduced number of bound lipids due to the steric restrictions that limit dendrimer deformation on the lipid bilayer [105]. Similar observations have been found for chemically modified dendrimers or different saturated and unsaturated lipid compositions of model membranes, as negatively charged lipids further strengthen dendrimer–membrane interactions [106,107].

**Figure 12:**
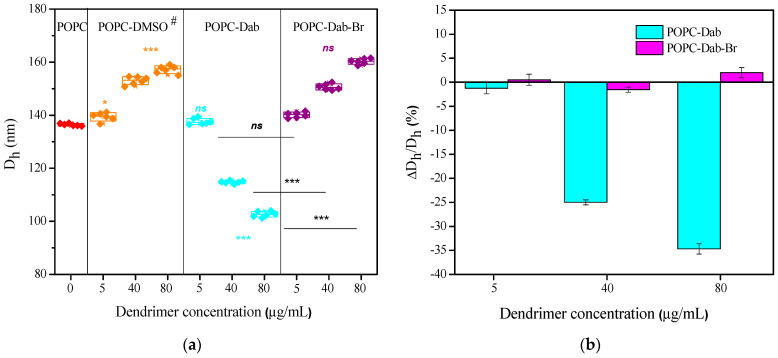
(**a**) Average vesicle size Dh (nm) and (**b**) relative change ∆Dh/Dh (%, panel (**b**)) of POPC vesicles as a function of dendrimer concentration (µg/mL). # designates DMSO volumes in µL corresponding to those of POPC-dendrimer suspensions: 0, 5, 40, and 80 µL DMSO. The data were analyzed with one-way ANOVA; *** and * denote *p* < 0.001 and *p* < 0.05 (colored stars compare POPC-dendrimers including DMSO, whereas dark ones compare pure POPC-dendrimers); *ns*: not significant data.

The size distribution of POPC LUVs was assessed by measuring the hydrodynamic diameter (Dh, nm) based on the dynamic light scattering approach. The original data for intensity size distributions provided by DLS experiments are presented in the Supplementary Data (SD). Figures S1 and S2 from SD illustrate the impact of the carrier, DMSO, and the dendrimers, Dab and Dab-Br dissolved in DMSO, on the average size distribution of POPC LUVs. Figure 12a and Table 1 provide a summary of the numerical data extracted from the graphs of Figures S1 and S2 (SD). The lowest concentrations of DMSO and DMSO-dissolved dendrimers did not affect the POPC vesicle size significantly. However, the higher DMSO and dendrimer concentrations resulted in considerable changes in the size distribution: whereas DMSO and Dab-Br increased the average POPC vesicle size with increasing concentrations, Dab exhibited an opposite effect (Figure 12). Considering the effect of DMSO on POPC LUVs’ size, it can be seen that Dab strongly decreased the vesicle size by 30%, unlike Dab-Br, where the average size was very close to the control conditions.

Furthermore, in Table 1, the polydispersity index (PDI) for DMSO (A) and DMSO-dissolved dendrimers (B) is summarized. In control POPC vesicles, PDI showed a value of 0.12, i.e., monodisperse POPC vesicle suspension. As the amount of DMSO or dendrimers increased, the monodispersity of POPC vesicles decreased. PDIs in DMSO and Dab-Br treated POPC vesicles were quasi-similar. However, in POPC-Dab vesicles, the polydispersity increased by 26%, considering the DMSO effect. To summarize, Dab decreased the average vesicle size and increased the polydispersity of LUVs’ suspension. However, the effect of Dab-Br could not be distinguished from those of DMSO, as both increased the vesicle size almost equally and had quasi-equal PDI at the highest studied concentrations of DMSO and DMSO-Dab-Br.

Our results correlate with those published elsewhere [107,108,109]. Other authors have demonstrated that carbosilane dendrimers did not change the average size of DMPC vesicles at physiological conditions with a ζ potential around 0 mV. In contrast, DMPC/DPPG and DPPG vesicle sizes increased dramatically with dendrimer concentration as ζ potential varied from −20 mV to +20 mV. These findings indicate that the dendrimers interact strongly with the negatively charged lipid bilayers and that the dendrimer–vesicle suspension forms aggregates. As expected, lipid composition is crucial in membrane–dendrimer interactions. These interactions did not affect vesicle size for neutral vesicles, whereas relatively large aggregates could be formed in vesicles with negatively charged surfaces. POPC and DMPC are both zwitterions, but POPC LUVs prepared in distilled water (pH 5.5) yield negative ζ potential (according to our data). In contrast, DMPC LUVs at physiological conditions (pH 7.4 and high salt concentrations) possess quasi-neutral ζ potential [108].

The treatment of POPC LUVs with Dab-Br did not significantly change the average size of the vesicles, and the effect was comparable to that of DMSO (Figure 12, Table 1). These results confirm our suggestion about the competition between the solvent and the dendrimer upon the interaction with the lipid molecules. A similar observation was reported by Trosheva et al. [110], who investigated the interaction between third-generation cationic pyridylphenylene dendrimers and multicomponent liposomes. The authors found no significant defects in the lipid liposomal surface upon dendrimer adsorption.

### 3.5. Visualization of Dendrimer Action on GUVs’ Morphology

**Figure 13:**
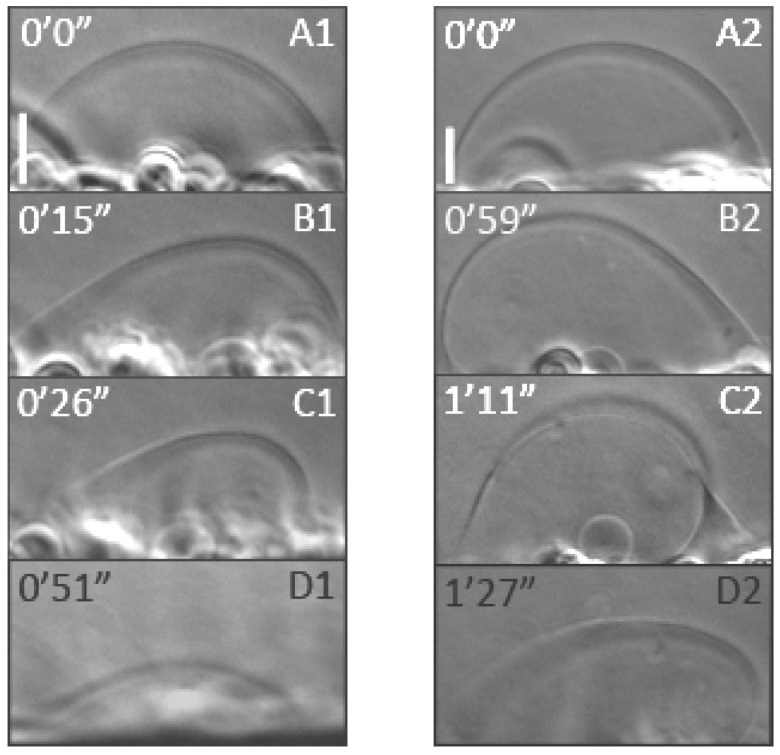
Phase contrast microscopy of GUVs composed of POPC (control, (**A1**,**A2**)), illustrating a time sequence of vesicle morphological changes induced by 6.6 µg/mL DMSO-dissolved Dab (**A1**–**D1**) and Dab-Br (**A2**–**D2**). Scale bar: 10 μm.

Giant unilamellar vesicles are an excellent model membrane system with size comparable to cells, which allows direct visualization under light microscope. They are widely used to study interactions between lipids and surface active molecules, like proteins, dendrimers, etc. [111,112,113]. Our experiments showed that after vesicle treatment with 5 µL Dab or Dab-Br (3.3 µg/mL in the working chamber), no visible morphological or size changes of GUVs were observed. The addition of another 5 µL of Dab or Dab-Br (6.6 µg/mL) resulted first in vesicle softening (visible membrane fluctuations) and then in gradual shrinking of POPC vesicle (Figure 13B1–D1,B2–D2). The dendrimers induced vesicle membrane disintegration and water leakage. The time of total vesicle disintegration was different depending on the dendrimer type: while in Dab-treated vesicles, the complete collapse was observed at about 1 min, Dab-Br did not result in such collapse. The effect of Dab dendrimer on POPC GUVs with similar size was studied on 12 vesicles, as the time for complete vesicle collapse on the electrodes was found to be 1.29 ± 0.45 min. Nine vesicles were treated with Dab-Br, and none collapsed for about 3 min at 6.6 µg/mL. The change of optical focus in the z-plane during the vesicle shrinking allowed the visualization of expulsed micron-scale aggregates, if any. Similar membrane disintegration was not observed, which means that membrane shrinking occurs by pore formation and expulsion of nanometric lipid–dendrimer aggregates.

Similar leakage of water from GUVs was observed under PAMAM G6 dendrimer treatment [96,111]. Authors have suggested that the rate of leakage depends on presence of charge lipids, dendrimer concentration, and its generation. Dendrimers can also alter the structure of small unilamellar vesicles [114] and supported lipid mono- [63,115] and bilayers [116,117,118]. The dendrimers caused these lipid model membranes to collapse once a specific critical surface concentration was reached. This collapse is akin to the morphological changes observed in red blood cells exposed to high dendrimer concentrations and appears to result from the stiffening of the cell membrane, as observed in our study [119,120,121]. As we demonstrated, the dendrimers Dab and Dab-Br interact with monolayers and bilayers and change the molecular organization of POPC membranes. At the concentrations studied, both dendrimers increased the order of the POPC membrane to a different extent, acting as a molecular “glue”, a term used by Sideratou et al. [122] for other dendrimers. This increase in molecular order likely originates from the observed decrease in area per lipid under Dab treatment as compared to the solvent, DMSO. Thus, Dab makes the membranes more rigid, which is consistent with Laurdan experiments. However, the effects of Dab-Br were difficult to differentiate from those of DMSO. Our data suggest that Dab-Br additionally increased the area per lipid in monolayer experiments, as observed with the studied DMSO concentrations. Some discrepancies were found regarding the impact of Dab-Br on POPC monolayers and vesicles, where both slightly softening (increased monolayer elasticity) and rigidifying (high membrane lipid order) effects on lipids were detected. It is important to note that the lipid–dendrimer aggregates exhibit different architectural forms depending on their stoichiometric ratio which, in turn, defines the physico-chemical properties of the membrane and its morphological transformations under dendrimer action. At low concentrations (5 μg/mL), the dendrimer easily adsorbs onto the membrane favored by ionic interactions [115,123]. A small number of dendrimer molecules are enough to change the zeta potential of the POPC membranes from negative to positive values. The significance of ionic interactions becomes more pronounced at the higher dendrimer concentrations and lower lipid/dendrimer ratio, which induce the vesicle disintegration through the shrinking of the membrane bilayer. The exact mechanism of action by which dendrimers disrupt membranes is rarely fully elucidated and remains a subject of ongoing debate. However, based on available data, surface interactions and pore formation are widely accepted as key components of their membrane-disrupting effects [119,120,121].

## 4. Conclusions

**Figure 14:**
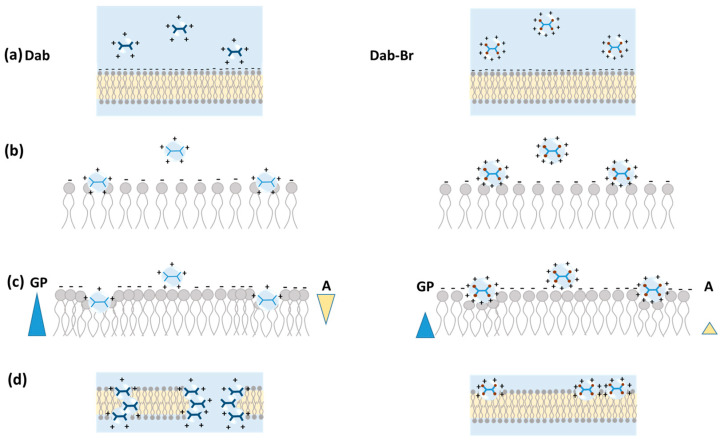
A potential molecular mechanism underlying the interactions between the dendrimers, Dab (left panel) and Dab-Br (right panel), and POPC molecules. (**a**) The positively charged dendrimers are attracted to the POPC membrane, which exhibits a negative zeta potential. (**b**) Adsorption of the dendrimers onto the membrane is facilitated by ionic interactions. (**c**) Dab penetrates into the unsaturated fatty acyl chains of POPC, whereas the larger and more highly charged Dab-Br molecule primarily interacts with the lipid polar head groups. These dendrimer–lipid interactions change the lipid membrane order (GP, general polarization) and molecular area (A). (**d**) Both dendrimers induce membrane shrinking, with Dab demonstrating a greater potency in disrupting the lipid membrane compared to Dab-Br.

This study evaluated the potential of two dendrimers, Dab and its brominated form Dab-Br, to interact with biological membranes for various biomedical applications. Several complementary biophysical approaches involving monolayers and vesicles were employed to reveal the mechanisms by which these dendrimers interact with POPC molecules. To summarize the obtained results, a potential molecular mechanism underlying the interactions between the studied dendrimers and the POPC membrane is illustrated in Figure 14. The proposed mechanism does not differentiate between dendrimer interactions with lipid monolayers and bilayers, as the results are consistent regardless of the experimental methods used. The interaction of the studied dendrimers with POPC molecules is supposedly dominated by the electrostatic mutual impact, which leads to a coupling between the polar groups of both molecular aggregates (Figure 14a,b). The surface pressure-area isotherms and compression modulus of the lipid monolayers studied before and after the addition of the dendrimers showed a different mechanism of interaction of Dab and Dab-Br with POPC. This could be explained by the assumption that Dab is likely to penetrate the unsaturated fatty acyl chains of POPC, leading to an alignment of the lipid tails, whereas the bigger and more positively charged molecule of Dab-Br interacts mainly into the lipid headgroup area (Figure 14c). This hypothesis was confirmed by FTIR-ATR experiments on the lipid molecular organization in the membrane in presence of the dendrimers. The reported spectral alterations support the conclusion that both dendrimer molecules perturb the lipid headgroup mobility. This effect is more pronounced for Dab in relation to its deeper impact on the membrane hydrocarbon region. Conversely, the interactions of Dab-Br with POPC appear to take place at the polar surface of the membrane as concluded from the spectral characteristics of the different POPC moieties’ vibrations.

The impact of dendrimers on membrane lipid order, ζ potential, and size distribution of LUVs comprising POPC was also assessed. Dab induced a higher lipid order in POPC vesicles compared to Dab-Br (Figure 14c), considering the fluidizing effect of the organic solvent. In addition, the dendrimers changed the negative ζ potential of POPC vesicles to positive values, indicating electrostatic interactions between the lipid headgroups and the dendrimers. Dab-Br exhibited a slightly higher ζ potential compared to Dab. However, Dab was able to reduce the average POPC vesicle size, unlike Dab-Br. These results confirm the postulated different mechanism of action of the dendrimers studied on model biomembranes.

Furthermore, the visualization of POPC GUVs revealed that the dendrimers induced vesicles’ concentration-dependent shrinking and complete disintegration, with Dab requiring lower concentration than Dab-Br to disrupt the membranes (Figure 14d).

In summary, the dendrimers Dab and Dab-Br exhibit a high potential to alter membrane characteristics, like area per lipid molecule, elasticity of the monolayer, lipid molecular organization, membrane order, zeta potential, vesicle size and stability, and polydispersity of the vesicle suspension. Our findings contribute to an in-depth understanding of the dendrimer–membrane interactions and highlight the potential of dendrimers to modulate membrane properties with regard to their biomedical applications.

## Figures and Tables

**Table 1:** Polydispersity index for control POPC LUVs treated with (**a**) DMSO and (**b**) dendrimers. Mean ± SD (n = 6).

(a)		(b)		
DMSO (µL)	POPC + DMSO	Dendrimers (µg/mL)	POPC + Dab	POPC + Dab-Br
0	0.12 ± 0.04	0	0.12 ± 0.04	0.12 ± 0.04
5	0.14 ± 0.02	5	0.14 ± 0.03	0.12 ± 0.02
40	0.16 ± 0.03	40	0.20 ± 0.03	0.17 ± 0.03
80	0.19 ± 0.02	80	0.24 ± 0.01	0.20 ± 0.04

## Data Availability

The original contributions presented in the study are included in the article, and further inquiries can be directed to the corresponding authors.

## Supplementary Materials

The following supporting information can be downloaded at: https://www.mdpi.com/article/10.3390/polym17070929/s1, Figure S1: Intensity size distribution of POPC LUVs as a function of the DMSO concentration. Same DMSO volumes in µL corresponding to those of POPC-dendrimer (Dab or Dab-Br) suspensions: 0, 5, 40 and 80 µL DMSO were studied. Figure S2: Intensity size distribution of POPC LUVs as a function of the dendrimer concentration, Dab or Dab-Br, dissolved in DMSO. Left column shows the changes in average size distribution of POPC LUVs under Dab tretatement (5, 40 and 80 µg/mL). Right column corresponds to Dab-Br treatement.
