# Supplementary material for: Molecular Mechanisms of Action of Dendrimers with Antibacterial Activities on Model Lipid Membranes
Source: Polymers (Basel). 2025 Mar 29;17(7):929. doi: 10.3390/polym17070929 (PMC11991017; doi:10.3390/polym17070929)
Supplement: Supplementary file 1 [file polymers-17-00929-s001.zip › polymers-3490387-supplementary.pdf]

Supplementary data

The original data for intensity size distributions provided by DLS experiments are presented below. Fig. S1 and S2 illustrate the impact of the carrier, DMSO, and the dendrimers, Dab and Dab-Br, dissolved in DMSO, on the average size distribution of POPC LUVs. Fig. 12a and Table 1 from the original paper provide a summary of the numerical data extracted from the graphs of Figures S1 and S2.

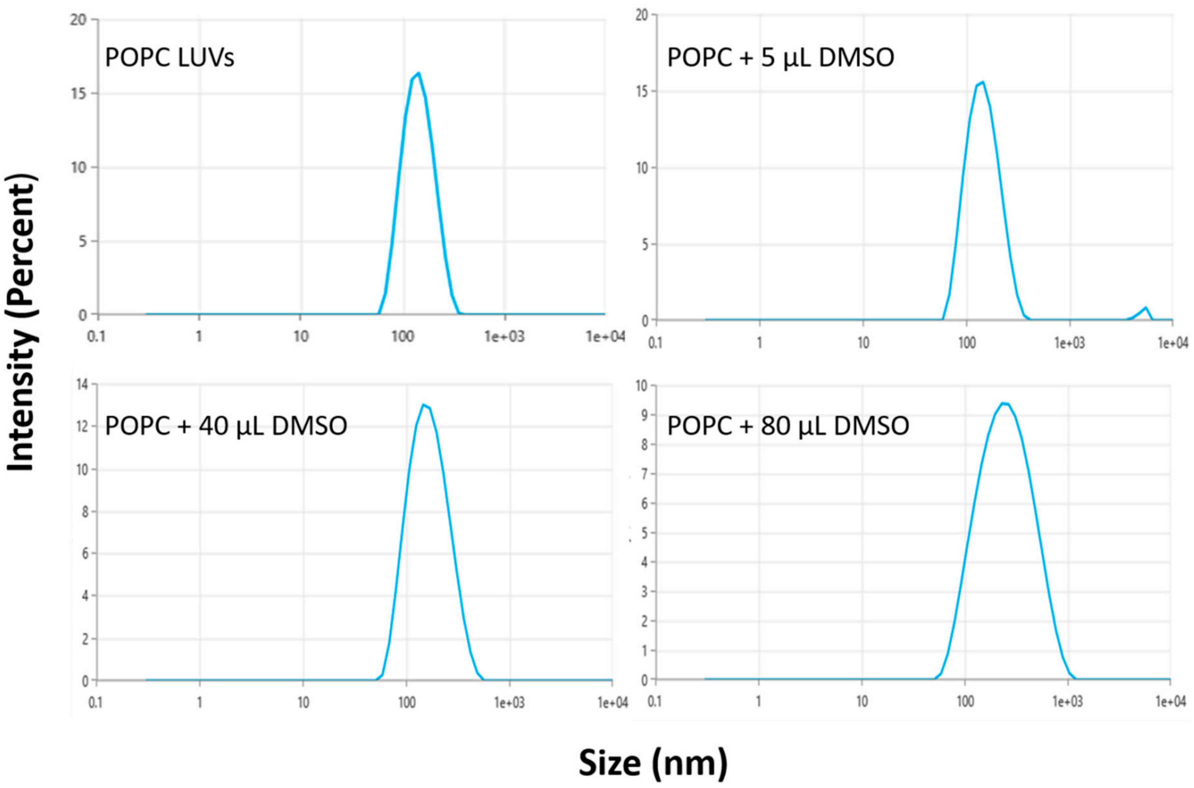

Figure S1. Intensity size distribution of POPC LUVs as a function of the DMSO concentration. Same DMSO volumes in µl corresponding to those of POPC-dendrimer (Dab or Dab-Br) suspensions: 0, 5, 40 and 80 µL DMSO were studied.

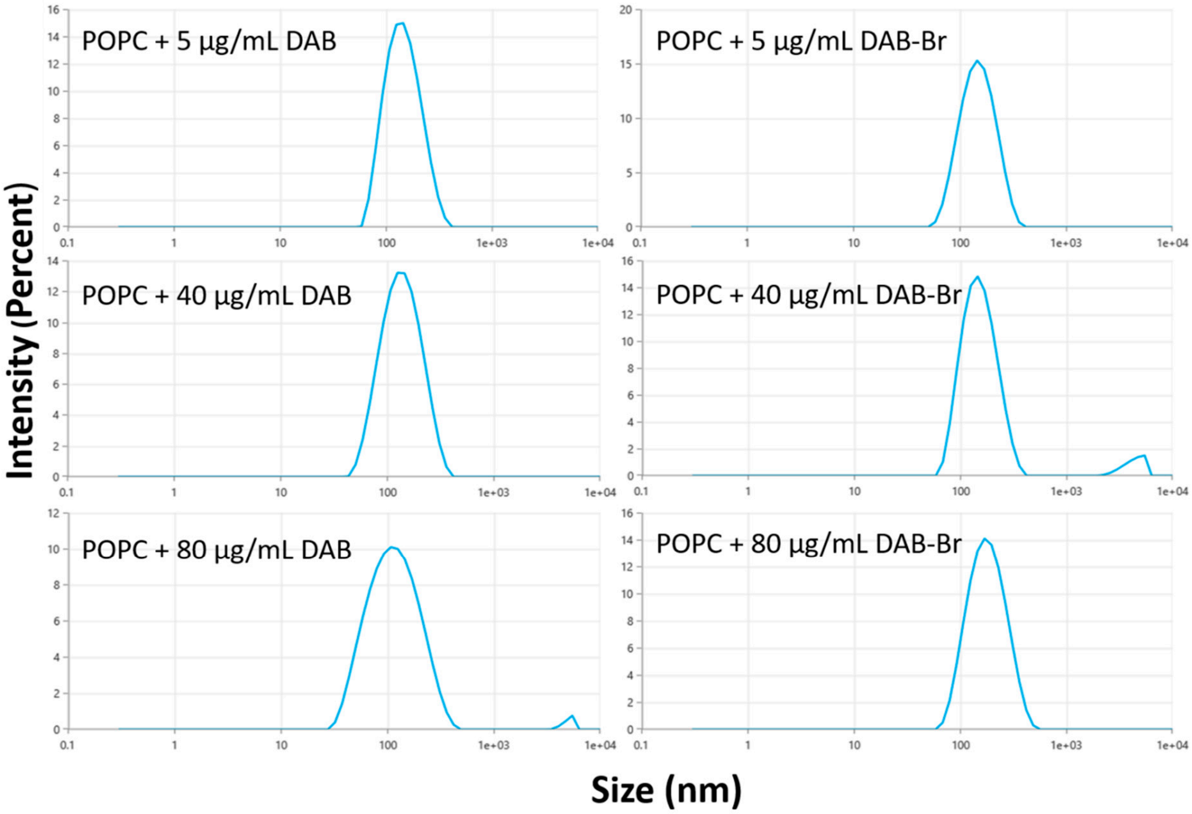

Figure S2. Intensity size distribution of POPC LUVs as a function of the dendrimer concentration, Dab or Dab-Br, dissolved in DMSO . Left column shows the changes in average size distribution of POPC LUVs under Dab tretatement (5, 40 and 80 µg/mL). Right column corresponds to Dab-Br treatement.
